# Using machine learning model explanations to identify proteins related to severity of meibomian gland dysfunction

**DOI:** 10.1038/s41598-023-50342-7

**Published:** 2023-12-22

**Authors:** Andrea M. Storås, Fredrik Fineide, Morten Magnø, Bernd Thiede, Xiangjun Chen, Inga Strümke, Pål Halvorsen, Hilde Galtung, Janicke L. Jensen, Tor P. Utheim, Michael A. Riegler

**Affiliations:** 1 Department of Holistic Systems, Simula Metropolitan Center for Digital Engineering, Oslo, Norway; 2 Department of Computer Science, OsloMet - Oslo Metropolitan University, Oslo, Norway; 3 The Norwegian Dry Eye Clinic, Oslo, Bergen, Norway; 4 Department of Ophthalmology, Sørlandet Hospital Arendal, Arendal, Norway; 5 Department of Plastic and Reconstructive Surgery, Oslo University Hospital, Oslo, Norway; 6 Department of Biosciences, University of Oslo, Oslo, Norway; 7 Department of Medical Biochemistry, Oslo University Hospital, Oslo, Norway; 8 Department of Ophthalmology, Vestre Viken Hospital Trust, Drammen, Norway; 9 Department of Computer Science, Norwegian University of Science and Technology, Trondheim, Norway; 10 Institute of Oral Biology, University of Oslo, Oslo, Norway; 11 Department of Oral Surgery and Oral Medicine, University of Oslo, Oslo, Norway; 12 Department of Ophthalmology, Oslo University Hospital, Oslo, Norway; 13 Department of Computer Science, UiT The Arctic University of Norway, Tromsø, Norway

**Keywords:** Machine learning, Protein analysis, Diagnostic markers, Computer science, Biomarkers, Corneal diseases

## Abstract

Meibomian gland dysfunction is the most common cause of dry eye disease and leads to significantly reduced quality of life and social burdens. Because meibomian gland dysfunction results in impaired function of the tear film lipid layer, studying the expression of tear proteins might increase the understanding of the etiology of the condition. Machine learning is able to detect patterns in complex data. This study applied machine learning to classify levels of meibomian gland dysfunction from tear proteins. The aim was to investigate proteomic changes between groups with different severity levels of meibomian gland dysfunction, as opposed to only separating patients with and without this condition. An established feature importance method was used to identify the most important proteins for the resulting models. Moreover, a new method that can take the uncertainty of the models into account when creating explanations was proposed. By examining the identified proteins, potential biomarkers for meibomian gland dysfunction were discovered. The overall findings are largely confirmatory, indicating that the presented machine learning approaches are promising for detecting clinically relevant proteins. While this study provides valuable insights into proteomic changes associated with varying severity levels of meibomian gland dysfunction, it should be noted that it was conducted without a healthy control group. Future research could benefit from including such a comparison to further validate and extend the findings presented here.

## Introduction

Meibomian gland dysfunction (MGD) is a condition characterized by an impairment of the physiologic function of the meibomian glands (MGs) and a reduced quantity or quality of the secreted meibum. Healthy meibum lowers the surface tension of the tear film and reduces ocular surface evaporation^1^. In patients with MGD, this loss of function increases tear evaporation, cause hyperosmolarity in the tears, and ocular irritations. MGD is one of the leading causes of dry eye disease (DED), affecting several hundred million people globally^2^. Both MGD and DED are tied to significantly impaired quality of life and substantial economic and social burdens^3,4^.

Despite the primary presentation of MGD being diminished function of the ocular surface lipid layer, it has long been thought that understanding the role of the tear film proteins may be key to understanding the etiology of MGD and related symptoms^5^. Despite the top 20 most abundant proteins accounting for around 90% of the total tear film proteins^6^, more than 1500 unique proteins have been identified in the tear film^7^, many of which likely have important bioactive and cellular signalling roles^8^.

Artificial intelligence, and in particular the subfield called machine learning (ML), is technology that can successfully solve a large variety of tasks in the field of ophthalmology^9^ and DED^10^. ML models learn from data to reach a predefined goal, such as estimating the tear film break-up time from clinical dry eye test results^11^. By exploring the inner workings of the resulting ML models, insights can be gained about which variables, also called *features*, in the dataset are important for the models when, e.g., making a diagnosis. This opens up for discovering new medical factors that are associated with medical conditions and diseases.

The aim of the current study was to explore potential relationships between the degree of MGD and protein expressions in tears and at the same time to build an ML pipeline. Emphasis was put on studying differences in tear protein expression across different severity levels of MGD, rather than simply comparing the proteomic profiles of healthy individuals to that of MGD patients. Techniques from ML were used to train predictive models and investigate what proteins the models regarded as important for predicting levels of MGD. Several of the proteins regarded as important by the models have previously been confirmed to be altered in the presence of DED by earlier studies. From a medical perspective, other insights might be gained by carefully investigating the proteins considered as important by the models. Some of the identified proteins might for example play a role in the pathogenesis of MGD and/or serve as potential targets for new treatments. They could also provide information about the severeness and prognosis of MGD.

## Methods

**Figure 1:**
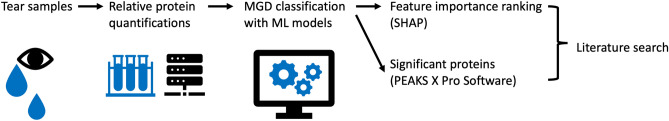
Overview of the workflow. Tear samples are extracted and the proteins quantified. Further on, ML models classify degree of MGD from the protein quantifications. The proteins are ranked by their importance in the models using Shapley Additive Explanations (SHAP), while PEAKS Pro X Software identify significant proteins. Identified proteins are investigated for potential relationship to MGD and DED through literature search.

This section describes the dataset, analysis methods, ML models and feature importance techniques applied in this work. A flowchart of the process is given in Fig. 1.

### Protein analysis and clinical tests

The dataset that the current work is based on includes detailed protein measurements from the tears of 320 patients with DED visiting the Norwegian Dry Eye Clinic during the years 2017 to 2019. No exclusion criteria were applied. The Regional Medical Ethics Committee of South-East Norway (REK) had approved the study (reference id 6892), and all medical procedures were performed in compliance with the Declaration of Helsinki. Written informed consent was given by all the patients. Since the present work focused on MGD, only the subset of patients with MGD were applied. As a result, the dataset in the current work included observations from 234 patients. Further descriptions about the tear protein collection, analysis and additional clinical tests for the original dataset are provided below.

Protein data is available from samples collected from Schirmer strips from the left eye of each patient. The quantity of tears equalled the full amount collected by the strips. The strips were stored in $500\,\upmu \text {l}$ phosphate buffered saline (PBS) and kept frozen until analyzed. Proteins were extracted from the strips using precipitation with cold acetone before trypsin digestion was performed. The tear proteins were then analyzed using untargeted liquid chromatography coupled to mass spectrometry (LC-MS) as previously described^12^. The PEAKS X Pro software was applied for relative protein quantification and protein identification as described earlier^13^: for label-free quantification (LFQ) using PEAKS X Pro, the following parameters were applied on protein level: false discovery rate (FDR) $\le 1\%$, fold change $\ge 2$, significance method analysis of variance (ANOVA) with at least 2 peptides. For normalization, the total ion current (TIC) was used. The LC-MS data was searched against the human Uniprot database (20384 entries) with PEAKS X Pro software version 10.5 (Bioinformatics Solutions, Waterloo, ON, Canada). The following parameters were used: digestion enzyme: trypsin, maximum missed cleavage: 1, fragment ion mass error tolerance: 0.05 Da and parent ion error tolerance: 10 ppm. Oxidation of methionine and acetylation of the N-terminus were specified as variable modifications. The maximum number of posttranslational modifications (PTMs) per peptide was set to 2. An FDR of 1% was applied to the datasets.

Additional results from clinical tests are available in the dataset applied in the current work, including fluorescein break-up time (FBUT), results from Schirmer test and the level of MGD. To measure FBUT, $5\,\upmu \text {l}$ 2% fluorescein was instilled with a micropipette and the seconds after a blink and before the tear film broke up was counted digitally. Unanesthetized Schirmer test was performed with sterile strips (0–30 mm/5 min) following standard protocols as described by Bron et al.^14^. Meibum quality and MG expressibility together with interpretation of meibography images were used to determine the severity level of MGD. To assess meibum quality and MG expressibility, a cotton swab was used to put a light pressure on the lower lid margin. Meibum quality was assessed according to a four-point scale (0: clear, 1: cloudy, 2: granular and 3: toothpaste), and a sum score for the central eight glands was calculated (range: 0–24). MG expressibility was graded on a four-point scale based on the number of expressible MGs among the central five glands (0: all glands expressible; 1: 3–4 glands expressible; 2: 1–2 glands expressible; 3: no glands expressible). Infrared (IR) meibography images were obtained using the Keratograph 5M (Oculus, Wetzlar, Germany). The amount of MG dropout was graded according to the Meiboscale by Pult^15,16^. The scale ranges from 0 to 4, where level 0 represents no glandular atrophy and the increasing grades represent increasing glandular loss. The MGD grading was performed as described in The International Workshop on Meibomian Gland Dysfunction^1^.

### Data preprocessing

Because the relative protein quantification dataset applied in the present work only includes one patient with MGD level 1, this patient was excluded. Consequently, the final dataset contained MGD grade data from 233 patients. Because tears were sampled from the left eye, the MGD level for the left eye was considered. This ensured that the tear proteins correspond to the severity of MGD in the same eye. The level of MGD ranged from level 2 to 4 with the majority of observations belonging to level 3. Patient characteristics are included in Table 1. From the table, it is observed that the majority of patients were female for all levels of MGD. The mean age tended to increase slightly with increasing severity of MGD even though the differences were not statistically significant. Further on, higher levels of MGD were associated with shorter FBUTs and higher values of meibum expressibility, meibum quality and MG dropout, showing statistically significant differences between the three MGD levels (*p* values $<0.05$). The significant differences are not unexpected because these clinical parameters are affected during MGD, and meibum expressibility and quality and MG dropout are all parameters included in MGD staging. The Schirmer test results were not statistically significantly different between the different levels of MGD.

**Table 1:** Characteristics of the patients included in the analyses.

MGD level	N (female/male)	Age (years)	OSDI	FBUT (s)	Schirmer (mm)	ME	MQ	MG dropout
2	31 (23/8)	51.9 (17.0)	32.0 (24.7)	5.1 (4.1)	15.5 (10.2)	1.5 (0.7)	6.0 (2.4)	2.1 (1.0)
3	126 (88/38)	54.3 (17.8)	27.4 (16.4)	4.5 (3.6)	14.7 (10.5)	2.0 (0.5)	8.3 (3.6)	2.4 (0.9)
4	76 (59/17)	55.8 (17.9)	34.3 (22.0)	3.3 (2.7)	14.4 (9.3)	2.7 (0.5)	11.1 (6.7)	2.8 (0.9)
Total	233 (170/63)	54.5 (17.7)	30.3 (19.8)	4.2 (3.5)	14.7 (10.1)	2.2 (0.6)	8.9 (5.0)	2.5 (1.0 )

Values are reported as mean with standard deviations in parentheses. Dry eye test results are for the left eye. ME, meibum expressibility; MQ, meibum quality; N, number of observations; OSDI, ocular surface disease index score.

From an ML perspective, input features that hold the identical value for all observations in the dataset do not provide any useful information to the model. Consequently, features representing proteins that were not identified in any of the patients, i.e., the feature values were ‘NULL’ for all observations, were removed from the dataset. Moreover, proteins with quantifications registered as 0 were replaced with 1. These proteins represent protein inference, meaning that the measurements do not necessarily belong to the protein of interest. Missing protein peak areas were replaced with 0, since missing values for the proteins mean that the proteins were not detected in the tear sample. The peak area should therefore be 0. Finally, proteins arising from contamination of the tear samples were removed prior to analysis. This includes keratins, except keratins 18 and 19, all dermcidins and dermacolines, as well as trypsin and putative trypsin-6. Most keratins, dermcidins and dermacolins are contaminations from the skin and should therefore not be included. Trypsin and putative trypsin-6 are proteins added to digest the proteins into smaller peptides. The final number of proteins included in the analysis was 2188. Logarithmic transformation followed by standard scaling was performed for the proteins prior to analysis due to wide value ranges and skewness in the value distributions of the relative protein quantifications.

### Machine learning analysis

A boosting tree-based ML algorithm called LGBMClassifier was trained to predict the level of MGD from the relative protein quantifications. The unbalanced numbers of patients in each MGD level was taken into account during training by setting the ‘is_unbalanced’ hyperparameter to *True*. To avoid overfitting, the ‘num_leaves’ hyperparameter was set to 2. For the remaining hyperparameters, default values were applied^17^.

First, an LGBMClassifier was trained to predict the MGD levels 2 to 4 from the relative protein quantifications. Next, the three MGD levels were predicted independently as three different binary classification problems. This was done to investigate if the different MGD levels relate to different proteins as features. Specifically, one model was trained to predict whether the patient had MGD level 2 or not, one model predicted whether the MGD level was 3 or not, and one model predicted whether the MGD level was 4 or not.

The full dataset was used to train the models, and model performance was calculated on the same dataset. The reason the data was not split into training and test sets was because the aim was to investigate relationships between protein expression and level of MGD rather than developing models for predictive purposes. By splitting the dataset, less information would be available for the models to learn the task. Due to class imbalance, model performance was evaluated using balanced accuracy, F1 score and Matthews correlation coefficient (MCC). For prediction of all MGD levels by the same model, the weighted average was used to calculate the F1 score.

### Feature importance

To investigate feature importances, Shapley Additive Explanations (SHAP)^18^ was applied. SHAP approximates Shapley values, which arrive from game theory and measure how much each feature contributes to the final model prediction^19^. Shapley values represent the fair share of the total payout (or model prediction) each player (or input feature) participating in a collaborative game should receive. Shapley values exhibit several attractive properties that make the methods based on these values popular for explaining ML models^20^. For instance, features that contribute equally to the model prediction receive the same Shapley value, while features that do not contribute receive a Shapley value of 0. The widely used library SHAP is based on the Shapley value and therefore shares the above-mentioned attractive properties^18^. It has established itself as a commonly used method for attributing importance of input features to ML models, including for tree-based models^21^, and this explanation method was chosen to explain the ML models in this study. The 15 most important features according to SHAP were examined for each of the four ML models. Because a low SHAP value represents a low feature importance for the model, the lowest-ranking features were not considered. The cutoff at 15 was chosen in order to focus on a limited number of proteins to investigate: the proteins represented by the 15 most important features according to SHAP were inspected in more detail by medical experts that are highly experienced in biomedical analyses, DED and MGD. Literature searches were performed by the experts for all the detected proteins to identify prior studies investigating the relevance of the proteins with respect to DED andMGD.

In addition to the original importance values provided by SHAP, a novel technique that weights the importance values by the model uncertainty was also proposed. This approach emphasizes feature importances from predictions the model is more certain about. The proposed weighting technique is outlined below.

For each observation in the dataset, the classification models output a probability score for each possible class. The probability score ranges from 0 to 1, where higher scores indicate that the models are more certain that the corresponding class is correct. Scores close to 0, on the other hand, mean that the models are certain that the corresponding class is not correct. If the probability scores are close to 0.5, the models are highly uncertain about which class the observation belongs to. The final model prediction is the class with the highest probability score. Based on the predicted probabilities, the predictions are divided into three groups: ‘Positive’, ‘Negative’ and ‘Borderline’. If the predicted class is correct and the corresponding predicted probability is $>0.6$, the predicted sample is ‘Positive’. However, if the predicted class is wrong, but the corresponding predicted probability is still $>0.6$, the predicted sample is ‘Negative’. All other predictions (correct and incorrect with predicted probabilities of 0.6 or below) are classified as ‘Borderline’. This is because the model is less certain about these predictions.

Next, the SHAP values for the corresponding predicted class are retrieved. If the sample is ‘Positive’, the SHAP value is increased according to the predicted probability as follows:

1 $$\begin{aligned} SHAP \, value_{weighted} = \frac{SHAP \, value_{original}}{1 - predicted \; probability} \end{aligned}$$

For negative samples, however, the SHAP values are decreased as follows:

2 $$\begin{aligned} SHAP \, value_{weighted} = SHAP \, value_{original} \cdot (1 - predicted \; probability) \end{aligned}$$

Because the predicted probabilities never exceed 1, this will result in reduced SHAP values for the wrong predictions and increased values for the correct predictions. In order to increase the SHAP value more as the predicted probability increases for the correct predictions, the value is divided by (1-predicted probability) rather than by the predicted probability directly. The same logic applies for the negative group. The SHAP values are not changed for the borderline group.

In the case of multiclass classification and correct class predictions, the SHAP values for the other classes are downweighted. Similarly, for incorrect predictions, SHAP values for the correct class are upweighted, while the wrong classes get their SHAP values downweighted. The weighting is performed as described above. Next, the sum of the SHAP values is calculated and the feature ranking is compared with the ranking of the original SHAP values. The weighted SHAP values are summarized in two different ways. First, samples from all three groups are included, i.e., the ‘Positive’, ‘Negative’ and ‘Borderline’ groups. As an alternative when no ground truth annotations are available, the SHAP values for borderline samples are simply excluded. This is because the model is less certain about these samples, and the feature importances for these samples can consequently be less reliable. The SHAP values for the rest of the samples are not changed. As for the model explanations using unweighted SHAP values, the proteins representing the 15 most important features following uncertainty weighting were examined by medical experts.

Finally, to compare the explainable artificial intelligence (XAI) methods with a more traditional approach for detecting proteins, an additional analysis using the PEAKS X Pro software was performed. This analysis identified the proteins that were significantly different between MGD levels 2, 3 and 4 and was described above in “Protein analysis and clinical tests” section.

### Technical details

Python version 3.9.2 was used for all experiments. Scikit-learn^22^ was used for data preparation and model evaluation, and lightgbm for training the LGBMCLassifier^17^. The SHAP library version 0.40.0 was used in the current study^18^. For reproducibility, the source code is made available online as described in Code availability.

## Results

### Machine learning models

When the classifier trained to predict MGD levels 2 to 4 was evaluated on the same data as used for training, the balanced accuracy was 72%, the F1 score was 0.82, and the MCC was 0.72. For the model predicting MGD level 2, the balanced accuracy, F1 score and MCC were 95%, 0.80 and 0.78, respectively. The corresponding model performance metrics for the model predicting MGD level 3 were 85%, 0.86 and 0.69, while for the model predicting MGD level 4, balanced accuracy, F1 score and MCC were 87%, 0.82 and 0.73, respectively.

### Unweighted feature importance

**Figure 2:**
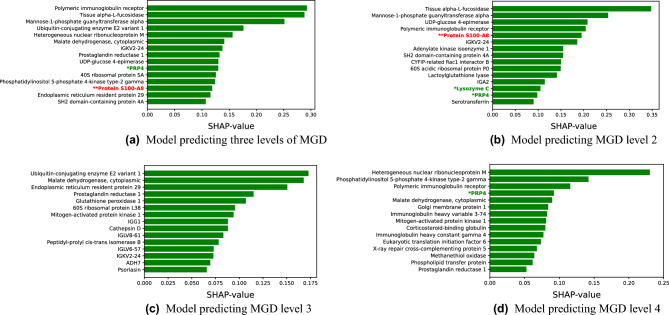
Unweighted feature importances for the four classifiers predicting MGD levels from relative protein quantifications using SHAP. *(green font): Protein identified as potential biomarker; downregulated in DED, **(red font): Protein identified as potential biomarker; upregulated in DED^8^. Figures are best viewed with zoom.

To determine which proteins were regarded as most important for predicting MGD severity, feature importance values were estimated for the final models. A comparison of the relative quantifications in MGD levels 2 to 4 for the proteins regarded as most important by the ML models are provided in Supplementary Table A.1. The importance plot for the model predicting all levels of MGD is shown in Fig. 2a. Higher values mean higher importance. When searching the literature, the features representing the proteins S100-A8 and proline-rich protein 4 (PRP4) are upregulated and downregulated in DED^8^. Several of the other proteins listed in Fig. 2a are also reported to be related to DED and MGD: The polymeric immunoglobulin receptor^23–26^, prostaglandine reductase 1^27^ and immunoglobulin kappa variable 2-24 (IGKV2-24)^28^.

Corresponding feature importance plots for the most relevant features for the three binary classification models are provided in Fig. 2b–d. For the model predicting MGD level 2 (Fig. 2b), the most important model feature represented tissue alpha-L fucosidase. Moreover, three of the other important features represented proteins that are upregulated (protein S100-A8) or downregulated (lysozyme C and PRP4) in DED^8^. Further on, features representing the immune system associated proteins IGKV2-24 and the polymeric immunoglobulin receptor were regarded as important features. These two proteins were also identified by the multiclass ML model. Serotransferrin and immunoglobulin alpha-2 heavy chain (IGA2) are additional proteins represented among the top features that are reported to be related to DED^6,29^.

For predictions of MGD level 3 (Fig. 2c), features representing ubiquitin-conjugating enzyme E2 variant and cytoplasmic malate dehydrogenase were regarded as most important. Prostaglandin reductase 1 and IGKV2-24 were also considered as important according to the SHAP values. Other highly ranked proteins that are reported as being related to DED are immunoglobulin lambda variable 8-61 (IGLV8-61)^30^, immunoglobulin lambda variable 6-57 (IGLV6-57)^30^, immunoglobulin gamma-1 heavy chain (IGG1)^31^, all-trans retinol dehydrogenase-7 (ADH7)^32,33^ and S100 calcium-binding protein A7, also known as psoriasin^34–36^.

Finally, for predictions of MGD level 4 (Fig. 2d), the feature representing heterogeneous nuclear ribonucleoprotein M was regarded as the most important feature. PRP4, which is downregulated in DED^8^, is also present in the figure. In addition, the features representing the polymeric immunoglobulin receptor and prostaglandin reductase 1 were considered important by the model according to SHAP.

### Weighted feature importance

The ranking of the proteins according to the weighted SHAP values are provided in Tables 2, 3, 4 and 5. Proteins that were not included in the corresponding feature importance plots using the original SHAP values are highlighted with bold font in the tables. For all the models, the feature rankings changed when the SHAP values were weighted. The relative quantifications in MGD levels 2 to 4 for the proteins are included in Supplementary Table A.1. Considering the multiclass classifier predicting all three levels of MGD, four of the top 15 most important features represented proteins that differ from the ranking with the original SHAP values. Three of these four proteins stood out as particularly interesting with respect to DED: glutathione peroxidase 1^37–39^, psoriasin and ADH7. For the classifier predicting MGD level 2, dynactin subunit 2, being a part of a complex that is involved in the secretion of proteins from cells in the lacrimal glands^40^, was one of the new highly ranked proteins. Regarding the MGD level 3 model, no new proteins were identified using weighted SHAP values, while for the MGD level 4 classifier, none of the new highest ranked proteins were found to be related to MGD or DED.

**Table 2:** Feature importances for the classifier predicting three levels of MGD from relative protein quantifications using SHAP weighted by model uncertainty.

Rank	Protein (accession number), incl. BL	Protein (accession number), excl. BL
1	Ubiquitin-conjugating enzyme E2 variant 1 (Q13404)	Polymeric immunoglobulin receptor (P01833)
2	Endoplasmic reticulum resident protein 29 (P30040)	Mannose-1-phosphate guanyltransferase alpha (Q96IJ6)
3	Polymeric immunoglobulin receptor (P01833)	Tissue alpha-L-fucosidase (P04066)
4	Tissue alpha-L-fucosidase (P04066)	Ubiquitin-conjugating enzyme E2 variant 1 (Q13404)
5	Malate dehydrogenase, cytoplasmic (P40925)	Heterogeneous nuclear ribonucleoprotein M (P52272)
6	Prostaglandin reductase 1 (Q14914)	Endoplasmic reticulum resident protein 29 (P30040)
7	Mannose-1-phosphate guanyltransferase alpha (Q96IJ6)	IGKV2-24 (A0A0C4DH68)
8	IGKV2-24 (A0A0C4DH68)	40S ribosomal protein SA (P08865)
9	**Glutathione peroxidase 1 (P07203)**	Prostaglandin reductase 1 (Q14914)
10	**IGG1 (P0DOX5)**	UDP-glucose 4-epimerase (Q14376)
11	Phosphatidylinositol 5-phosphate 4-kinase type-2 gamma (Q8TBX8)	Phosphatidylinositol 5-phosphate 4-kinase type-2 gamma (Q8TBX8)
12	Heterogeneous nuclear ribonucleoprotein M (P52272)	PRP4 (Q16378)
13	**Psoriasin (P31151)**	Protein S100-A8 (P05109)
14	**ADH7 (P40394)**	SH2 domain-containing protein 4A (Q9H788)
15	PRP4 (Q16378)	Malate dehydrogenase, cytoplasmic (P40925)

**Bold font**: Not among the original top ranked features. BL, borderline predictions.

**Table 3:** Feature importances for the classifier predicting MGD level 2 from relative protein quantifications using SHAP weighted by model uncertainty.

Rank	Protein (accession number), incl. BL	Protein (accession number), excl. BL
1	Tissue alpha-L-fucosidase (P04066)	Tissue alpha-L-fucosidase (P04066)
2	Mannose-1-phosphate guanyltransferase alpha (Q96IJ6)	Mannose-1-phosphate guanyltransferase alpha (Q96IJ6)
3	UDP-glucose 4-epimerase (Q14376)	UDP-glucose 4-epimerase (Q14376)
4	IGKV2-24 (A0A0C4DH68)	Protein S100-A8 (P05109)
5	Polymeric immunoglobulin receptor (P01833)	Polymeric immunoglobulin receptor (P01833)
6	Protein S100-A8 (P05109)	IGKV2-24 (A0A0C4DH68)
7	CYFIP-related Rac1 interactor B (Q9NUQ9)	SH2 domain-containing protein 4A (Q9H788)
8	60S acidic ribosomal protein P0 (P05388)	Adenylate kinase isoenzyme 1 (P00568)
9	Adenylate kinase isoenzyme 1 (P00568)	60S acidic ribosomal protein P0 (P05388)
10	SH2 domain-containing protein 4A (Q9H788)	Lactoylglutathione lyase (Q04760)
11	Lactoylglutathione lyase (Q04760)	CYFIP-related Rac1 interactor B (Q9NUQ9)
12	IGA2 (P0DOX2)	IGA2 (P0DOX2)
13	**Dynactin subunit 2 (Q13561)**	Lysozyme C (P61626)
14	Lysozyme C (P61626)	PRP4 (Q16378)
15	**Alpha-centractin (P61163)**	Serotransferrin (P02787)

**Bold font**: Not among the original top ranked features. BL, borderline predictions.

**Table 4:** Feature importances for the classifier predicting MGD level 3 from relative protein quantifications using SHAP weighted by model uncertainty.

Rank	Protein (accession number), incl. BL	Protein (accession number), excl. BL
1	Ubiquitin-conjugating enzyme E2 variant 1 (Q13404)	Ubiquitin-conjugating enzyme E2 variant 1 (Q13404)
2	Endoplasmic reticulum resident protein 29 (P30040)	Endoplasmic reticulum resident protein 29 (P30040)
3	Malate dehydrogenase, cytoplasmic (P40925)	Malate dehydrogenase, cytoplasmic (P40925)
4	Glutathione peroxidase 1 (P07203)	Prostaglandin reductase 1 (Q14914)
5	Cathepsin D (P07339)	Glutathione peroxidase 1 (P07203)
6	60S ribosomal protein L38 (P63173)	Mitogen-activated protein kinase 1 (P28482)
7	Prostaglandin reductase 1 (Q14914)	60S ribosomal protein L38 (P63173)
8	IGG1 (P0DOX5)	Cathepsin D (P07339)
9	Mitogen-activated protein kinase 1 (P28482)	IGLV8-61 (A0A075B6I0)
10	Peptidyl-prolyl cis-trans isomerase B (P23284)	IGG1 (P0DOX5)
11	IGLV6-57 (P01721)	Peptidyl-prolyl cis-trans isomerase B (P23284)
12	IGLV8-61 (A0A075B6I0)	IGLV6-57 (P01721)
13	IGKV2-24 (A0A0C4DH68)	ADH7 (P40394)
14	ADH7 (P40394)	IGKV2-24 (A0A0C4DH68)
15	Psoriasin (P31151)	Psoriasin (P31151)

BL, borderline predictions.

**Table 5:** Feature importances for the classifier predicting MGD level 4 from relative protein quantifications using SHAP weighted by model uncertainty.

Rank	Protein (accession number), incl. BL	Protein (accession number), excl. BL
1	Heterogeneous nuclear ribonucleoprotein M (P52272)	Heterogeneous nuclear ribonucleoprotein M (P52272)
2	Phosphatidylinositol 5-phosphate 4-kinase type-2 gamma (Q8TBX8)	Phosphatidylinositol 5-phosphate 4-kinase type-2 gamma (Q8TBX8)
3	Polymeric immunoglobulin receptor (P01833)	Polymeric immunoglobulin receptor (P01833)
4	Golgi membrane protein 1 (Q8NBJ4)	Golgi membrane protein 1 (Q8NBJ4)
5	**Corticosteroid-binding globulin (P08185)**	PRP4 (Q16378)
6	PRP4 (Q16378)	Immunoglobulin heavy variable 3-74 (A0A0B4J1X5)
7	Immunoglobulin heavy variable 3-74 (A0A0B4J1X5)	**Corticosteroid-binding globulin (P08185)**
8	Immunoglobulin heavy constant gamma 4 (P01861)	Mitogen-activated protein kinase 1 (P28482)
9	Malate dehydrogenase, cytoplasmic (P40925)	Malate dehydrogenase, cytoplasmic (P40925)
10	X-ray repair cross-complementing protein 5 (P13010)	Immunoglobulin heavy constant gamma 4 (P01861)
11	Mitogen-activated protein kinase 1 (P28482)	Eukaryotic translation initiation factor 6 (P56537)
12	Phospholipid transfer protein (P55058)	Methanethiol oxidase (Q13228)
13	Eukaryotic translation initiation factor 6 (P56537)	X-ray repair cross-complementing protein 5 (P13010)
14	Methanethiol oxidase (Q13228)	Phospholipid transfer protein (P55058)
15	**Protein NDRG1 (Q92597)**	Prostaglandin reductase 1 (Q14914)

**Bold font**: Not among the original top ranked features. BL, borderline predictions.

### Label-free quantification using PEAKS

According to the PEAKS analysis of the protein quantifications for the patients with MGD levels 2–4, 13 proteins were found to be significantly different between the three levels of MGD. The proteins are listed in Supplementary Table A.2. Among these, thymidine phosphorylase has been associated with DED^41,42^. Supplementary Table A.2 also reports the changes in relative protein quantifications between the three different levels of MGD for the significant proteins.

### Summary of the findings

**Figure 3:**
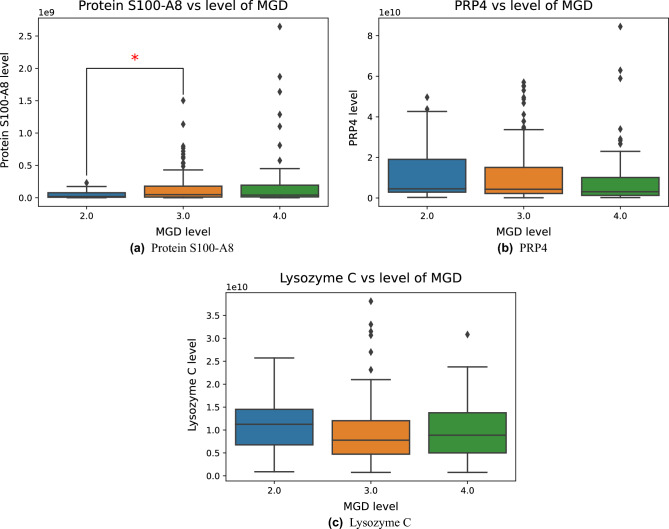
Relative quantifications of protein S100-A8, PRP4 and lysozyme C for different levels of MGD. Statistically significant changes in protein levels (*p* values $<0.05$) are marked with *.

To summarize, several of the most important features in each of the four ML models represented proteins that are known to be upregulated or downregulated in DED. One of these was protein S100-A8, which is upregulated in DED^8^. A boxplot of the relative quantifications of protein S100-A8 for the different MGD levels in our dataset is provided in Fig. 3a. It is observed from the plot that the protein levels were mostly stable, but for the higher MGD levels, the levels of protein S100-A8 were skewed towards higher values. The differences in protein quantifications were statistically significant (*p* value $<0.05$) between MGD levels 2 and 3, while the differences were borderline significant between MGD levels 2 and 4 (*p* value = 0.052). PRP4 is downregulated in DED^8^. Figure 3b plots PRP4 quantifications for the MGD levels. Even though the plot shows a trend toward higher values for MGD levels 2 and 3 compared to level 4, the differences were not statistically significant. Since PRP4 has been found not to be significantly downregulated in lipid deficient dry eye, which includes MGD, this might explain why the levels were more stable in this plot^43^. Finally, relative quantifications of lysozyme C, which is known to be downregulated in DED^8^, are plotted for different levels of MGD in Fig. 3c. The protein amounts tended to be higher in tears from patients with MGD levels 2 compared to level 3 and 4. However, no statistically significant changes were found. The significant protein differences for protein S100-A8 support that this proteins might play a central role in MGD. Still, because several of the MGD level groups expressed similar protein levels, it suggests that the ML models also identified patterns that go beyond alterations in single protein quantifications. Corresponding fold changes in the relative quantifications of protein S100-A8, PRP4 and lysozyme C between MGD levels 2, 3 and 4 are included in Supplementary Table A.1.

## Discussion

The work presented in this paper shows that ML models combined with the feature importance method SHAP are able to detect proteins that are relevant for DED and MGD. Because one aim of the study was to explore proteomic changes in subjects with increasing severity of disease, the models were trained on patients with MGD only. Among the top 15 ranked features in each of the four ML models, there were several proteins that are interesting with respect to DED and MGD. These proteins are PRP4, S100 calgranulin A8, lysozyme C, prostaglandine reductase 1, psoriasin, serotranferrin, ADH7, the polymeric immunoglobulin receptor, and the immunoglobulins IGKV2-24, IGLV8-61, IGLV6-57, IGA2 and IGG1. Their relationships to DED and MGD are discussed below.

Jackson et al.^8^ performed a literature review about proteins in DED and identified some proteins that are promising biomarkers for DED. Three of the proteins detected with the method proposed in the current study were found to be potential biomarkers that are upregulated or downregulated in DED according to the review: PRP4, S100 calgranulin A8 and lysozyme C. These three proteins are marked in Fig. 2a–d. Compared to healthy controls, the expression of PRP4 and S100 calgranulin A8 were significantly different in both aqueous-deficient and combined aqueous-deficient and evaporative DED, while lysozyme C was only significantly differentially expressed in combined aqueous-deficient and evaporative DED^6^.

Further on, prostaglandin reductase 1 is involved in the breakdown of prostaglandins^44^. Prostaglandins trigger inflammatory responses on the ocular surface and were considered as a contributor to the pathogenesis of DED^27^. Prostaglandin reductase 1 might therefore be a key protein with respect to DED and MGD.

Psoriasin, being associated with the immune system and inflammation, has been found to be present at high levels in MGs from individuals without any diseases affecting or involving the lacrimal glands^34^. Moreover, the gene for psoriasin was upregulated in diseased MGs^35^ as well as in saliva from patients with primary Sjögren syndrome (pSS)^36^. It should, however, be noted that all included subjects in the latter study were females. Taken together, psoriasin could be of value for the grading of MGD and serve as a potential biomarker for DED.

Serotransferrin binds and transports iron and exhibits antimicrobial effects by keeping iron unavailable to pathogens^45^. MGD is associated with microbial infections. Serotransferrin was increased in aqueous deficient and combined aqueous deficient and evaporative DED^6^. Moreover, levels of serotransferrin increased with increasing age^46^. Since the risk of experiencing DED also increases with increasing age, this indicates a potential relationship between serotransferrin and DED. Also considering its antimicrobial effects and that the protein levels were altered in several types of DED, serotransferrin might play a role in the pathogenesis of of MGD.

ADH7 is part of a class IV alcohol dehydrogenase primarily involved in the oxidation of retinol to retinaldehyde and possibly retinoic acid synthesis^47,48^. Retinoids are associated with the proliferation, differentiation, keratinization and apoptosis of corneal epithelial cells and deficiency of vitamin A can cause both DED and keratopathy^32^. A metabolite of vitamin A, 13-cis retinoic acid, is known to cause MGD and has been shown to alter gene expression related to inflammation and differentiation as well as inducing apoptosis of MG epithelial cells in vitro^33^.

The polymeric immunoglobulin receptor was significantly downregulated in patients with aqueous-deficient DED compared to individuals without DED^23^. The protein was also decreased in tears from rabbits with Sjögren syndrome-associated dry eye^24^. Moreover, the protein was suppressed in reflex tears, that are produced in response to irritant stimulations^25^. Huang et al. found, on the contrary, that the polymeric immunoglobulin receptor was upregulated in tears from patients with DED^26^. Despite contradicting findings, changes in the protein expression have been detected in different types of DED, suggesting that it could play a role in the pathogenesis of the disease. Further on, IGA2 is important for the adaptive immune response and binds to the polymeric immunoglobuline receptor^29^. Amorim et al.^28^ found the levels of IGKV2-24 to be significantly higher in tears from patients with proliferative diabetic retinopathy compared to non-diabetic controls. In this study, $74 \%$ of the proliferative diabetic retinopathy patients exhibited Schirmer test results $< 10$ mm/5 min, and tear film breakup-times were significantly reduced compared to the controls. In our dataset, FBUT was significantly lower for patients with MGD level 4 compared to MGD levels 2 and 3. Consequently, the importance of IGKV2-24 in our models could also be affected by differences in FBUT between the patient groups. Still, considering their roles in the immune response and the fact that MGD facilitates microbial infections in the eye^49^, IGKV2-24’s, IGA2’s and the polymeric immunoglobulin receptor’s roles in MGD could be investigated further.

Both IGLV8-61 and IGLV6-57 are immunoglobulins involved in the immune response. In a study investigating tear proteomics following laser-assisted in-situ keratomileusis (LASIK) and small incision lenticule extraction (SMILE) surgery, IGLV8-61 and IGLV6-57 were both upregulated one week after LASIK surgery^30^. The patients had a mean tear film break-up time of 6.1 and Schirmer test of approximately 9 mm/5 min, which are clinical signs of DED. Even though the observed alterations in IGLV8-61 and IGLV6-57 probably arose from the surgery, they could also be connected to dry eye-related pathology.

IGG1, also involved in the adaptive immune response, could play a role in the development of dry eyes. Mackie et al. reported increased tear levels of immunoglobulin gamma in patients with DED compared to healthy controls^31^. A study comparing allergic conjunctivitis in mice with and without DED observed significantly increased levels of IGG1 regardless of DED, suggesting that DED did not affect the protein expression^50^. However, since allergic conjunctivitis activate the adaptive immune response, this might mask possible alterations during DED. A reduction of IGG1 in Descemet’s membrane, which is a part of the cornea, was observed for patients with Fuchs endothelial corneal dystrophy^51^. Taken together, alterations of IGG1 expression are observed for several diseases on the ocular surface, including DED.

When weighting the SHAP importance values by the model uncertainty, two additional proteins that are potentially important for MGD were identified. These proteins were glutathione peroxidase 1 and dynactin subunit 2. Moreover, psoriasin, IGG1 and ADH7 were ranked among the top 15 features for the multiclass model, which was not the case without uncertainty weighting. The relevance of glutathione peroxidase 1 and dynactin subunit 2 with respect to MGD and DED is discussed below.

Studies have shown that oxidative stress plays a role in the mechanism of DED^52^. Glutathione peroxidase 1 controls the levels of reactive oxygen species (ROS) and is important for the normal function of the MGs^37^. Hyperosmolarity, which is often observed with DED and pSS are both associated with reduced levels of this protein^38,39^. Consequently, glutathione peroxidase 1 stands out as a potentially promising biomarker for MGD.

Dynactin subunit 2 is a part of the dynactin complex, which binds to and activates dynein. Further on, this facilitates exocytosis from acinar cells, which reside in the lacrimal glands and secrete proteins onto the ocular surface^40^. Studies of mouse models for pSS indicate that the protein secretion from acinar cells are altered independently of inflammatory responses^53,54^. Future research should therefore look into whether human lacrimal gland secretion is affected by changes in levels of the dynactin subunit 2 and whether such changes are associated with MGD and DED.

Regarding the significant proteins detected by the PEAKS X Pro software, thymidine phosphorylase is potentially relevant for DED and MGD. Thymidine phosphorylase is associated with endothelial cell survival and function^41^. Loss-of-function has been reported to give dry eyes in a case report, although as part of a complex systemic clinical picture^42^. Further research is required to investigate the potential role of thymidine phosphorylase in MGD.

Remarkably, only one of the significant proteins detected by PEAKS Pro X, mannose-1-phosphate guanyltransferase alpha, was also found by the feature importance approach. Consequently, it seems like these two approaches complement each other, and applying both of them can result in a higher number of identified potential biomarkers for diseases.

When using the feature importance approaches, most of the proteins identified as promising biomarkers for MGD are involved in the immune response and inflammation. Other proteins, such as PRP4, ADH7 and dynactin subunit 2, contribute to the normal function of the eye. These findings confirm that MGD is a complex condition associated with disturbance of processes in the healthy eye and activation of several inflammatory and immunologic pathways. There is most likely not one single biomarker, but rather a panel of biomarkers, that characterize MGD.

Even though several of the important features identified in this study represented proteins that were previously found to be related to DED, the majority of the features did not. Highly ranked proteins such as mannose-1-phosphate guanyltransferase alpha, 60S ribosomal proteins L6 and L13 and corticosteroid-binding globulin have not been extensively studied with respect to DED and MGD. However, mannose-1-phosphate guanyltransferase alpha is associated with disease in general, and the other proteins are involved in the regulation of inflammation. Investigating the relevance of these and other highly ranked proteins with respect to DED and MGD might give rise to novel medical discoveries.

Some of the detected proteins that are potential biomarkers for MGD, for example psoriasin and the polymeric immunoglobulin receptor, are altered in pSS. It is not unreasonable that some proteins can play key roles in both MGD and pSS, especially because pSS has dry eyes as a primary symptom and has been associated with MGD^55,56^. Still, the most upregulated tear protein in pSS, neutrophil gelatinase-associated lipocalin^57^, was not among the detected proteins in the current study. One possible explanation is that patients with MGD make up a more diverse group, also including patients without pSS. Even though MGD and pSS can occur simultaneously, these are two separate diagnoses, and it is expected that there are differences in how they affect the ocular surface and tear composition.

The feature importance plots differed between the MGD models. This is not surprising, as we expect different protein levels to be of higher or lower importance according to the severity of the MGD. Because MGD is associated with inflammation, one would for example expect proteins related to inflammation to be ranked differently for the different levels of MGD. Still, many of the same proteins were highly ranked for all models. This means that there were also similarities between the different levels of MGD.

The reliability of the estimated feature importance values will be affected if the ML models are not performing well. In this work, the balanced accuracy for the multiclass ML model was 72%, while the balanced accuracies for the three binary ML models were 85% or higher. The high model performances indicate that the predicted important features should be robust. At the moment, there exists no method that takes the model uncertainty into account when determining the feature importance. In the current work, the estimated importance values were weighted based on the predicted probabilities as a mean of including uncertainty into the feature ranking. When the ground truth annotations are available, the importance values for correct predictions with high certainty from the model can be assigned more weights than incorrect and/or uncertain predictions. By following this, a change in the feature ranking was observed, and new potentially MGD-related proteins were identified. However, when the ground truth is not available, the correct model predictions cannot be identified. In this case, only the original importance values for the most certain model predictions were included. Using this technique, no new proteins were present among the 15 most important features. This is probably due to the low number of uncertain predictions. The results indicate that the weighting is most effective when the ground truth is known.

The dataset applied in this study has several strengths. First, it includes a high number of patients. Moreover, the comprehensive proteomic analyses provide detailed information about the tear composition for the patients and were combined with several clinical parameters including level of MGD. Finally, because all patients were examined at the same eye clinic, variations between the performance of the various procedures are expected to be minimal. Taken together, these strengths are adding reliability to the reported results.

This study has some potential limitations. First, the ML models were not evaluated using an external test set. As a result, the models’ abilities to generalize to new patients are not known. The main reason for not dividing the dataset into training and test sets was because the aim was to describe patterns in the data rather than developing ML models for predictive purposes. This approach is motivated by a method called ‘microscope artificial intelligence (AI),’ where models are developed and explained in order to gain a deeper understanding of the data used to train the models^58,59^. Similar to applying a microscope for studying our surroundings in more detail, explaining the ML model can enable us to view the data from a different angle, potentially leading to new discoveries. According to microscope AI, the goal is to extract knowledge out of the data rather than creating models for automatic decision-making^58^. Still, the ML models from the current work should not be used for diagnostic purposes since they have not been externally evaluated.

Another potential limitation is the chosen tear collection method. The tears in the present study were collected using Schirmer strips, adhering to previous protocols presented by our group^57,60^. However, the optimal methodology for collecting tear samples remains an area of debate. Collection methods such as microcapillary tubes with or without saline flush, Schirmer strips as well as the intrinsic individual variability may contribute to both composition and concentration of collected proteins. Some studies found similar results regarding protein expression following collection with microcapillary tubes and Schirmer strips^61,62^, while another study indicates that the collection method might impact the proteins detected in the sample^63^. In the present project, unanesthetized Schirmer tests were conducted, which can stimulate reflex tearing. Indeed, at least 15 proteins have been noted to differ between reflex and basal tears^25^, where basal tears might be regarded as more relevant when studying MGD. However, it can be argued that the previous studies mentioned above indicate that the applied collection method using Schirmer strips ensures a representative collection of biological material as well as a low degree of contamination. Moreover, although an anesthetized Schirmer test might be more representative concerning basal tears, the topical anesthesia might serve as a contaminant and alter tear composition.

DED is broadly divided into aqueous deficient DED and evaporative DED^64^. The latter is the more common of the two and MGD is the most common cause of evaporative DED. These subdivisions of DED are not mutually exclusive. Instead, they often overlap along a spectrum referred to as mixed DED, which is commonly seen in the clinic. In the MGD dataset used in the current work, some of the patients with MGD also exhibit signs of aqueous deficient DED. This might be a potential limitation because some proteins have been noted to be altered as a result of lacrimal tear production^65^, which further on can give rise to differences in protein measurements between individuals with and without aqueous deficient DED. On the other hand, the fraction of patients with Schirmer test values $< 5$ mm was relatively low and stable for each of the MGD levels 2 to 4, ranging between 16 and $22\%$. Consequently, if the protein measurements were affected due to reduced tear production, it would most likely have similar effects across all these MGD levels. The inclusion of patients with mixed DED probably had little effect on the reported results. Still, it would be interesting to conduct studies specifically targeting tear proteomic patterns in patients with mixed aqueous deficient DED and evaporative DED. While the dataset in the present work only includes 46 out of 233 patients with mixed aqueous deficient DED and MGD, data collection from a larger cohort is necessary to get reliable results for this subgroup of DED patients. Future work should look into this topic.

This study explored which proteins are regarded as important for ML models predicting different levels of MGD. Several of the detected features represented proteins that from earlier research are known to be altered in DED. What might seem unusual with the presented work is that a control group without MGD was not included for training the ML models. However, the aim was not to distinguish healthy eyes from eyes diagnosed with MGD, but rather to explore the expression of proteins in tears for different levels of MGD. Individuals without MGD were not regarded as relevant to include in the training dataset because they might obscure the proteomic differences within the MGD patient group. However, future work should look into how the current results compare to a control group without MGD. Investigating the protein rankings in healthy controls can strengthen the findings in the present study.

In conclusion, this study successfully combined ML and proteomics to explore relationships between MGD severity and protein expression in tears. Examination of which proteins the ML models regarded as important for predicting levels of MGD showed that several of the highest ranked proteins are known to be upregulated or downregulated in DED. Other proteins that might be relevant in MGD were also detected. Future work should explore these proteins further with the aim to increase the understanding of MGD and develop improved treatment options. Moreover, the proposed method for detecting potentially relevant proteins could be applied to other types of medical conditions and diseases with the aim to discover new medical knowledge.

### Supplementary Information

Supplementary Information.

## Data Availability

The data is currently not publicly available since it contains sensitive patient data. The interested reader is encouraged to contact the corresponding author if access to the data is needed.

## Code availability

The code is made publicly available and can be accessed through the following link: https://github.com/AndreaStoraas/MGD-proteins-XAI.
