# Supplementary material for: Using machine learning model explanations to identify proteins related to severity of meibomian gland dysfunction
Source: Sci Rep. 2023 Dec 22;13:22946. doi: 10.1038/s41598-023-50342-7 (PMC10746717; doi:10.1038/s41598-023-50342-7)
Supplement: Supplementary file 1 — Supplementary Information. [file 41598_2023_50342_MOESM1_ESM.pdf]

Using machine learning model explanations to identify proteins related to severity of meibomian gland dysfunction

Andrea M. Storås, Fredrik Fineide, Morten Magnø, Bernd Thiede, Xiangjun Chen, Inga Strümke, Pål Halvorsen, Hilde Galtung, Janicke L. Jensen, Tor P. Utheim, Michael A. Riegler

Table A.1. Fold changes in relative protein quantifications for the proteins detected as most important by the ML models, listed in alphabetical order. level: MGD level, NA: not available.

Protein (accession number)	Fold changes (level 2: level 3: level 4)
40S ribosomal protein SA (P08865)	1.00 : 0.91 : 1.48
60S acidic ribosomal protein P0 (P05388)	1.00 : 0.96 : 0.95
60S ribosomal protein L38 (P63173)	1.00 : 1.71 : 1.40
Adenylate kinase isoenzyme 1 (P00568)	1.00 : 0.82 : 0.78
Adenylyl cyclase-associated protein 1 (Q01518)	1.00 : 1.18 : 1.03
ADH7 (P40394)	1.00 : 1.14 : 0.84
Alpha-centractin (P61163)	1.00 : 1.11 : 0.97
Cathepsin D (P07339)	1.00 : 1.22 : 1.27
Corticosteroid-binding globulin (P08185)	1.00 : 2.83 : 1.88
CYFIP-related Rac1 interactor B (Q9NUQ9)	1.00 : 0.91 : 0.95
Dynactin subunit 2 (Q13561)	1.00 : 1.27 : 0.70
Endoplasmic reticulum resident protein 29 (P30040)	1.00 : 1.38 : 1.06
Eukaryotic translation initiation factor 6 (P56537)	1.00 : 1.00 : 0.85
Glutathione peroxidase 1 (P07203)	1.00 : 1.11 : 0.96
Golgi membrane protein 1 (Q8NBJ4)	1.00 : 2.75 : 1.57
Heterogeneous nuclear ribonucleoprotein M (P52272)	1.00 : 1.36 : 0.72
IGA2 (P0DOX2)	1.00 : 1.28 : 1.83
IGG1 (Q95M34)	NA
IGKV2-24 (A0A0C4DH68)	1.00 : 0.76 : 1.11
IGLV6-57 (P01721)	1.00 : 1.96 : 2.13
IGLV8-61 (A0A075B6I0)	1.00 : 0.82 : 1.13
Immunoglobulin heavy constant gamma 4 (P01861)	1.00 : 0.71 : 1.57
Immunoglobulin heavy variable 3-74 (A0A0B4J1X5)	1.00 : 1.03 : 1.23
Lactoylglutathione lyase (Q04760)	1.00 : 1.06 : 1.21
Lysozyme C (P61626)	1.00 : 0.89 : 0.70
Malate dehydrogenase, cytoplasmic (P40925)	1.00 : 1.00 : 0.92
Mannose-1-phosphate guanyltransferase alpha (Q96IJ6)	1.00 : 2.36 : 2.32
Methanethiol oxidase (Q13228)	1.00 : 1.26 : 1.03
Mitogen-activated protein kinase 1 (P28482)	1.00 : 0.97 : 0.93
Peptidyl-prolyl cis-trans isomerase B (P23284)	1.00 : 0.97 : 0.83
Phosphatidylinositol 5-phosphate 4-kinase type-2 gamma (Q8TBX8)	1.00 : 1.44 : 1.18
Phospholipid transfer protein (P55058)	1.00 : 1.33 : 0.96
Polymeric immunoglobulin receptor (P01833)	1.00 : 1.09 : 1.37
Prostaglandin reductase 1 (Q14914)	1.00 : 0.88 : 0.71
Protein NDRG1 (Q92597)	1.00 : 1.25 : 0.82
Protein S100-A8 (P05109)	1.00 : 2.08 : 2.86
PRP4 (Q16378)	1.00 : 0.78 : 0.59
Psoriasin (P31151)	1.00 : 0.87 : 2.44
Serotransferrin (P02787)	1.00 : 1.32 : 1.02
SH2 domain-containing protein 4A (Q9H788)	NA
Tissue alpha-L-fucosidase (P04066)	1.00 : 1.00 : 1.00
Ubiquitin-conjugating enzyme E2 variant 1 (Q13404)	1.00 : 0.88 : 0.99
UDP-glucose 4-epimerase (Q14376)	1.00 : 1.92 : 1.04
X-ray repair cross-complementing protein 5 (P13010)	1.00 : 1.47 : 1.16

Table A.2. Fold changes in relative quantifications for the proteins found to be significantly altered between patients with MGD levels 2, 3 and 4, respectively using the PEAKS X Pro software. The protein ranking is based on significance value: Higher values receive higher ranks. level: MGD level.

Rank	Protein (accession number)	Fold changes (level 2: level 3: level 4)
1	S-methyl-5'-thioadenosine phosphorylase (Q13126)	1.00 : 2.38 : 1.21
2	60S ribosomal protein L6 (Q02878)	1.00 : 2.02 : 1.86
3	Mannose-1-phosphate guanyltransferase alpha (Q96IJ6)	1.00 : 2.09 : 1.78
4	Programmed cell death protein 10 (Q9BUL8)	1.00 : 1.60 : 2.29
5	Eukaryotic peptide chain release factor GTP-binding subunit ERF3A (P15170)	1.00 : 1.62 : 2.00
6	Proteasome subunit alpha type-7 (O14818)	1.00 : 1.82 : 2.07
7	V-type proton ATPase catalytic subunit A (P38606)	1.00 : 2.62 : 1.63
8	Complement C1q subcomponent subunit B (P02746)	1.00 : 2.01 : 1.70
9	60S ribosomal protein L13 (P26373)	1.00 : 2.44 : 1.25
10	Thymidine phosphorylase (P19971)	1.00 : 2.48 : 2.53
11	Protein Niban 2 (Q96TA1)	1.00 : 1.85 : 2.68
12	T-complex protein 1 subunit eta (Q99832)	1.00 : 2.33 : 1.18
13	GMP reductase 2 (Q9P2T1)	1.00 : 2.62 : 1.78
